# Promising methods for detection of novel coronavirus SARS‐CoV‐2

**DOI:** 10.1002/viw2.4

**Published:** 2020-03-23

**Authors:** Ran Liu, Aisi Fu, Zixin Deng, Yan Li, Tiangang Liu

**Affiliations:** ^1^ Key Laboratory of Combinatorial Biosynthesis and Drug Discovery Ministry of Education and Wuhan University School of Pharmaceutical Sciences Wuhan China; ^2^ Wuhan Dgensee Clinical Laboratory Co., Ltd. Wuhan China; ^3^ State Key Laboratory of Microbial Metabolism Joint International Research Laboratory of Metabolic & Developmental Sciences, and School of Life Sciences and Biotechnology Shanghai Jiao Tong University Shanghai China; ^4^ Hubei Engineering Laboratory for Synthetic Microbiology Wuhan Institute of Biotechnology Wuhan China; ^5^ Department of Clinical Laboratory Renmin Hospital of Wuhan University Wuhan China

**Keywords:** coronavirus, COVID‐19, early diagnosis, nucleic acid detection, SARS‐CoV‐2, 2019‐nCoV

## Abstract

A very recent outbreak of the novel coronavirus, COVID‐19, in the city of Wuhan, China, in December 2019 and its subsequent spread within and across China have resulted in several deaths and infections. Presently, nucleic acid amplification test is essential for the confirmation of COVID infection. In this report, we summarized the six promising methods, including whole‐genome sequencing, real‐time reverse transcription polymerase chain reaction, nanopore target sequencing, antibody‐based immunoassay techniques, use of paper‐based biomolecular sensors, and the clustered regularly interspaced short palindromic repeats‐Cas system‐based technology, which can also be deployed for the detection of SARS‐CoV‐2. We further introduced the principles of these methods, discussed the scope and practicability of application of the available products and methods, and highlighted the potential approaches to develop additional products and techniques for early diagnosis of COVID‐19.

## Graphical abstract

The development of antibody‐based immunoassay or nucleic acid testing for detection of SARS‐CoV‐2 infection has become critical for COVID‐19 outbreak control. We introduced the principles of these methods, discussed the scope and practicability of application of the available products and methods, and highlighted the potential approaches to develop additional products and techniques for early diagnosis of COVID‐19.

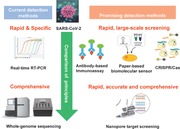

An ongoing outbreak of the novel coronavirus (COVID‐19) pneumonia that hit the city of Wuhan, China, in December 2019 subsequently began to spread to other provinces/regions of China and also to other countries worldwide. As of February 6, 2020, 28 275 people were confirmed with SARS‐CoV‐2 infection, and 24 702 people were suspected to be infected.^1^ Usually, this newly identified coronavirus has an incubation period of 2‐7days,^2^ and there are no obvious symptoms that are observed during the incubation period. However, the virus is contagious and can spread from an infected person to a non‐infected person during the incubation period. Nucleic acid (NA) testing is reported to be essential for the confirmation of SARS‐CoV‐2 infection. Therefore, in this report, we have summarized some newly developed and promising methods to detect SARS‐CoV‐2 (Figure 1), in order to facilitate the development of novel approaches for early diagnosis.

**FIGURE 1:**
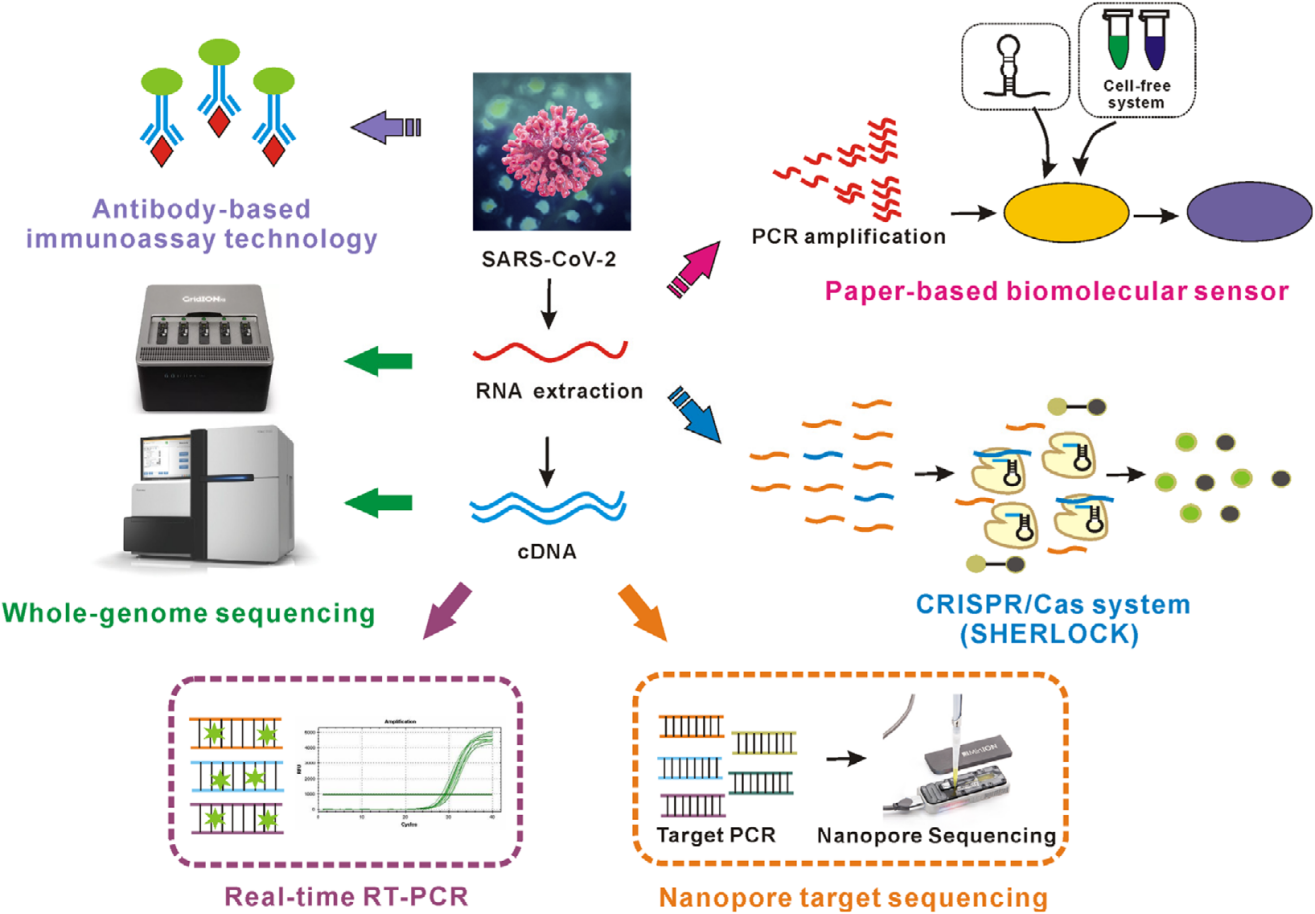
Developed and promising methods for detection of novel coronavirus SARS‐CoV‐2

Whole‐genome sequencing is one of the most comprehensive approaches for the identification of the viral NAs. The first complete SARS‐CoV‐2 genome sequence was uploaded on GenBank (accession number MN908947) by Zhang et al. on January 5, 2020. Five typical open reading frames (ORFs) were identified in the SARS‐CoV‐2 genome: ORF1ab polyprotein (7096 amino acids), spike glycoprotein (1273 amino acids), envelope protein (75 amino acids), membrane protein (222 amino acids), and nucleocapsid protein (419 amino acids). As of February 6, 2020, 19 other genome sequences of SARS‐CoV‐2 obtained by using Illumina or Nanopore platforms were also uploaded on GenBank by researchers in China,^3^, ^4^ the United States, and Australia. As whole‐genome sequencing is relatively expensive, time consuming, and complicated, it is unsuited for urgent and large‐scale testing.

Real‐time reverse transcription polymerase chain reaction (RT‐PCR) is the most popular testing method for the detection of SARS‐CoV‐2. In this method, the SARS‐CoV‐2 RNA is first reverse transcribed into cDNA, and specific gene fragments within the cDNA are amplified using target‐specific primers. The fluorescence signal, which represents the copy number of the target sequence, can be easily detected during the amplification process. The U.S. Centers for Disease Control and Prevention (CDC) recommended to use 2019‐nCoV_N1, 2019‐nCoV_N2, and 2019‐nCoV_N3 primers and probes that target the nucleocapsid gene,^5^ whereas the Chinese CDC recommended to use the ORF1ab and nucleocapsid primers as the targets for the detection of SARS‐CoV‐2.^6^ Real‐time RT‐PCR is specific, rapid, and economical; however, by using this approach we cannot precisely analyze the NA sequence of the amplified gene fragments, and thus all the target fragments that are successfully amplified are considered to be positive. In addition to these limitations, sample preparation, laboratory conditions, and technical errors can lead to false‐negative results. Also, several reports have revealed false‐negative results obtained by using real‐time RT‐PCR kits. Besides, testing for viral NAs using this method must be carried out only by experienced technicians in qualified laboratories, making its practical applications by clinicians limited.

In our laboratory we combined the advantages of target amplification and real‐time nanopore sequencing to develop a novel method, which is designated as nanopore target sequencing (NTS; unpublished data). At the initial step, this method involves the amplification of several SARS‐CoV‐2‐specific gene fragments. These targets were chosen based on the virulence genes and were not limited to sites currently recommended by the Chinese and the U.S. CDCs, allowing us to enhance the sensitivity of this novel assay. We then used the nanopore platform to perform the sequencing of the amplified fragments. Thus, the nanopore platform can sequence as well as analyze the results simultaneously, further allowing us to confirm whether the tested sample possess SARS‐CoV‐2 within a few minutes （at the fast speed） after sequencing. The results of this experiment revealed the detailed NA sequences and can thus reflect whether the virulence genes were mutated during the spread of the virus, thereby providing substantial information for further epidemiological analysis. To detect the presence of other respiratory viruses causing infections in patients, we added multiple characteristic primers for amplification of common respiratory viruses including conventional coronavirus, influenza A virus, influenza B virus, influenza C virus, parainfluenza virus, respiratory syncytial virus, adenovirus, rhinovirus, metapneumovirus, and boca virus. Therefore, this single NTS assay is capable of comprehensively and simultaneously analyzing and diagnosing various types of viral infections.

Antibody‐based immunoassay techniques combined with different signal detection methods are also found to be typically rapid detection methods. For example, immune colloidal gold detection technique can yield positive results that are visible to the naked eye within a few minutes of testing. Test strips that are developed by using the immune colloidal gold technology can be easily used in the home settings and are also suitable for large‐scale testing. Although some test strips have been developed using the antibody‐based immunoassay technique, no reports of the identification of SARS‐CoV‐2‐specific antibodies have been published yet. Therefore, the specificity and sensitivity of this method need to be further investigated to be used as a potential detection method.

Use of the programmable RNA sensors is another promising approach for RNA virus detection. Green et al. have developed toehold switch sensors that bind to and can sense any RNA sequence virtually.^7^ Toehold switch sensors are mainly composed of two cognate RNAs: a transducer RNA that encodes for the output signal of the system (e.g., green fluorescent protein mRNA) and a trigger RNA that modulates the output signal. The output signal represses translation by sequestering the ribosomal binding site (RBS) of the transducer RNA within a hairpin. This hairpin is unwound upon binding of a cognate trigger RNA, exposing the RBS and enabling the translation of the downstream protein. Researchers have so far used a freeze‐dried, paper‐based, and cell‐free protein expression platform for the deployment of these toehold switch sensors outside of a research laboratory, which have provided a sterile and abiotic method for the storage and distribution of these genetic circuits at room temperature. Initially, an ∼36 nucleotide long, specific RNA sequence for detection of Ebola virus or Zika virus was designed as the trigger RNA, and this system was used to develop a rapid, and low‐cost detection method.^8^, ^9^ As SARS‐CoV‐2 is also an RNA virus, therefore, the same system could theoretically be used to develop a rapid detection method for the coronavirus.

The clustered regularly interspaced short palindromic repeats (CRISPR)‐Cas system is used in another promising method for NA detection. Cas13a (previously known as C2c2)^10^ can be effectively reprogrammed with CRISPR RNAs to provide a platform for specific RNA sensing. Upon recognition of its RNA target, activated Cas13a engages in “collateral” cleavage of the nearby nontargeted RNAs.^11^ Zhang and Collins' laboratories have collaborated to develop the SHERLOCK method that is based on the CRISPR/Cas13a system.^12^ This method uses the reverse transcriptase recombinase polymerase amplification coupled with T7 transcription to amplify the target RNA fragments of Zika virus or Dengue virus. These target RNA sequences further activate Cas13a, and the activated Cas13a then cleaves the reporter RNA (quenched fluorescent RNA) allowing for real‐time detection of the target RNA. Therefore, this SHERLOCK method could theoretically be developed as a rapid detection method for SARS‐CoV‐2.

All the above‐described methods can be effectively deployed for the diagnosis of COVID‐19 in different settings. For instance, real‐time RT‐PCR is still the most widely used detection method in large hospitals, whereas antibody‐based immunoassay techniques, paper‐based biomolecular sensors, and the CRISPR‐Cas system‐based methods (e.g., SHERLOCK) are still expected to be further developed as large‐scale screening methods that can even be used in the home settings. Moreover, NTS and whole‐genome sequencing are the most comprehensive detection methods. Also, NTS combines sensitivity, comprehensiveness, rapid detection, and low cost, making it the most suitable method for the detection of suspected viral infection that cannot be diagnosed effectively by other methods.

## CONFLICT OF INTEREST

There is no conflict of interest to declare.
